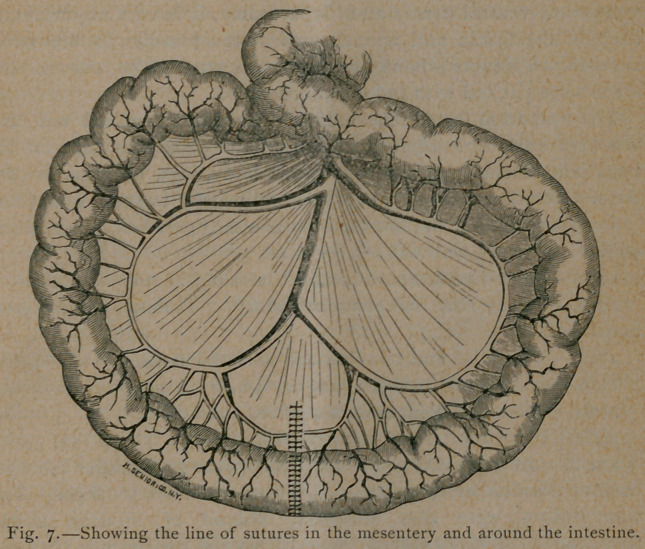# Laparotomy and Intestinal Suture

**Published:** 1887-05

**Authors:** John A. Wyeth

**Affiliations:** Professor of Surgery in the New York Polyclinic; visiting surgeon to Mt. Sinai Hospital, etc.


### LAPAROTOMY AND INTESTINAL SUTURE.

### EXSECTION OF A PORTION OF THE ILEUM, WITH RESTORATION OF FUNCTION OF THE ALIMENTARY CANAL BY SUTURE.*

*By John A. Wyeth, M. D., Professor of Surgery in the New York Polyclinic; visiting surgeon to Mt. Sinai Hospital, etc.*

LEAH R,† Russian, fifty-six years old, housewife, was admitted to Mt. Sinai Hospital on October 9, 1886, with the following history: For ten years she had a swelling in the left groin, which would disappear when she lay down and return when she was standing erect. She had not worn a truss. Two weeks before admission she discovered that the tumor no longer disappeared upon going to bed, but became painful, tender, and more swollen. She had not vomited up to the time of arriving at the hospital, but there had been no evacuation of the bowels for six days prior to her admission.

On admission, a swelling as large as an ordinary fist was found occupying the inner aspect of the left groin and thigh. The skin over the tumor was red in color, tender and doughy to the touch, and fluctuation was evident. The tissues around were slightly emphysematous. The patient's appetite was gone; she was emaciated, having lain in her present condition ten days in a tenement-house without proper care. The temperature was normal.

A diagnosis of strangulated femoral hernia was made, ether administered, and the tumor incised. Several ounces of foul pus mixed with intestinal matter were discharged. No trace of a hernial sac or intestine could be discovered, such was the gangrenous condition of the mass. Upon introducing the little finger into the femoral canal, a slight opening into the intestine could be felt.

* Read before the Section in Surgery of the New York Academy of Medicine, March 14, 1887.

† I am indebted to Dr. Rich, of the house-staff of Mt. Sinai Hospital, for the notes of this case.

Into this closed dressing forceps was introduced, and the opening dilated by separating the jaws of this instrument. This was intended to secure the freer exit of ingested matter from the upper portion of the occluded gut.

A loose dressing of iodoform gauze was laid over the wound. The patient improved in condition after this operation, under mild stimulation and liquid diet (milk, beef-tea, beef-juice, whisky, sherry, etc.) Only a small quantity of ingested matter escaped when the gauze dressing was changed on every second or third day.

On October 22d, thirteen days after the first operation, with ether narcosis laparotomy was performed. The patient was placed upon the back with the pelvis elevated upon a firm cushion. With Volkmann's spoon the granulation tissue was first scraped from the walls of the abscess, the hole into the intestine plugged with a pellet of iodoform gauze, the cavity of the abscess irrigated with 1–to-1,000 sublimate, and then tightly packed with iodoform gauze.

The integument about the femoral canal was washed thoroughly with soap and warm water, cleanly shaved, washed with ether, and finally with 1–to–1,000 sublimate solution. Towels wrung out of hot sublimate solution (1–to–3,000) were laid over that portion of the body near the groin, leaving only a spot exposed measuring six by four inches.

An incision four inches in length was made parallel with the outer border of the rectus muscle, the lower end being over the *femoral ring*. All bleeding was arrested, so that before the peritonæum was opened the wound was absolutely dry. Juniperized catgut ligatures were employed. Great care was observed to keep to the inner side of and away from the epigastric vessels which were exposed in the dissection. The parietal layer of the peritonæum was picked up with a fine forceps, opened, and further divided upon the finger as a director.

Upon looking into the abdominal cavity, one or two loops of normal small intestine were seen, and, upon displacing these upward, a third loop was seen to be imprisoned in the femoral opening. That part of this loop above the constriction was slightly distended, while the part on the side nearest the rectum was contracted until it was about two thirds of the diameter of the upper segment. The obstruction of the intestinal canal at the ring was complete. A soft flat sponge taken from a warm Thiersch solution

(boric acid, gr. iv; salicylic acid, gr. j; water, ℥j) was placed beneath the imprisoned loop in such a manner that it held the loose loops of small intestine back, and was ready to receive any foreign matter which might escape from the gut when it was divided.

Two long-jawed scissors-forceps (used as clamps) were then placed so as to close the loop of gut which was caught in the ring. One of these rested against the inner surface of the ring, and the other only sufficiently removed from this to permit of a division of the intestine between the forceps.

As soon as this was effected, the loose end, with one pair of forceps attached, was brought out through the abdominal wound and placed in a warm Thiersch towel. As the forceps which

Fig. 1.—Loop of small inthstine. *a b*, lines of section through the gut, removing the gangrenous portion; *b c*, same through the mesentery; *a a*, gangrenous portion of ileum; *d d*, occlusion of the afferent and efférent tubes by tape ligaturcs.

constricted the ring of gut attached to the femoral canal was removed, a tuft of sponge was tightly packed into this ring to prevent any infection from the abscess with which it cummunicated.

Of the loop which had been liberated, about ten inches (five above and below the point of occlusion) were drawn out of the abdomen, flat Thiersch sponges carefully placed so as to close the wound and prevent any escape of matter into the peritoneal cavity, and the exposed gut protected by covering with warm towels. A piece of cotton tape one fourth of an inch wide was then tied four inches above and below the limits of the gangrenous opening, so as to completely occlude the lumen of the gut (*d d*, Fig. 1). These tapes had been well soaked in a 1-to-3,000 sublimate solution. When the forceps-clamp was removed, the opening into the intestine was seen to occupy two thirds of the circumference of the canal. The gut was then cut across at a right angle to its axis by a single stroke with the straight scissors (*a b*, Fig. 1). These lines of section were well out in sound tissue. The piece of intestine removed measured two inches and a half. A triangular piece of the mesentery was also removed (*b c b*, Fig. 1).

The bleeding from the mesentery was profuse, requiring a dozen catgut ligatures. From the ends of the intestine only a slight oozing occurred. The cavity of the gut from the tapes to the openings was carefully emptied of all matter and washed out with Thiersch's solution. Nothing escaped from the lower end.

The edges of the divided mesentery were first united by eight interrupted catgut sutures about one fourth of an inch distant from each other. When the intestine was reached, the mesenteric attachment of each end was carefully brought into apposition and the work of stitching the ends of the cylinders to each other begun.

In doing this, three forms of suture were employed :

1. A suture through the mucous membrane alone, or *Czerny's suture*. 2. That through the peritoneal coat alone, or *Lembert's suture*. 3. One which pierces the peritoneal coat and, passing along with the muscular layer, comes out on the free border of the divided gut, the *intermediate suture.**

In Fig. 2, which represents a longitudinal section through the ends to be approximated, is shown at *b* the Czerny suture as it is passed through the mucous layer of the gut from the inner sur-

* Dr. Sutton, of Pittsburgh, employed this suture in a case which ended in good recovery. I saw the line of union in this patient about two years after the operation, through the courtesy of Professor J. B. Hunter, who was performing a second laparatomy.

face of the canal, while at *a* the method of introducihg the Lembert suture through the peritoneal layer is shown.

Fig. 2.—Schematic. *a*, Lembert's, and *b*, Czerny's sutures.

When a gut is cut across, the longitudinal muscular layer retracts, carrying the peritoneal layer with it and leaving the thick mucous membrane projecting about one eighth of an inch. The object of the Czerny suture is to bring the mucous membrane and the connective tissue upon which it rests together, and thus strengthen the line of union after adhesion occurs. If this is not done, the slight adhesion between the peritoneal surfaces obtained by the Lembert suture might give way under the strain of distension of the intestine by gas or ingested matter. The objection to passing a suture entirely through the wall of the gut and thus approximating all the coats at once is the danger that the perforation may be followed by escape of gas or other contents to either side of the line of adhesion between the ends. The inversion of the mucous membrane by Czerny's suture and of the peritoneal layer by Lembert's suture after the threads are tied is shown in Fig. 3.

Fig 3.—Schematic. Showing the inversion of the peritoneal layer by tying Lembert's suture, and of the mucous membrane by Czerny's suture.

The mechanism of the intermediate suture is well shown in Fig. 4. This suture adds strength to the union by taking in the muscular layer and connective tissue of the mucous membrane together with the peritoneal covering. Applied after the Czerny suture, there can be no danger of escape of intestinal contents through the wound.

In suturing the intestine, the very finest black (iron-dyed) silk, and a delicate, perfectly round needle, should be used. The straight needles are preferable to those which are half or full curved. The thread should be made aseptic in sublimate solution (1 to 3,000) and it and the needle taken from a 1-to-20 carbolic-acid solution as they are used.

Fig. 4.—Schematic. Showing the route of the intermediate sutures.

In commencing the sutures, first insert one Czerny suture just over the mesenteric or attached border of the intestine, and tie this, the knot, of course, coming within the lumen of the gut. The needle should pass from within through the mucous layer at a distance of about three sixteenths of an inch from the free border (Fig. 2), out along the free border of the same end, and, being carried across to the opposite end, should be made to enter below the muscular and mucous layer, and to emerge through the mucous layer three sixteenths of an inch from its cut edge. A Lembert suture should be next inserted just at the edge of the mesenteric attachment as follows :* The needle is made to enter the peritoneal coat one eighth of an inch from the edge, and passing between the serous and mucous coats, is again brought through the peritoneal layer about one twenty-fifth of an inch from the edge (Fig. 2, *a*). At a point exactly opposite, the same stitch is passed through the peritoneal layer of that side for the same distance, and this thread is tied. In knotting all of these sutures it is a wise precaution to use the *double* or *friction* knot for the first tying, for by so doing there is no danger of the suture slipping and the parts separating as the second turn is being made. A second Lembert suture should now be inserted on the other side of the mesenteric attachment, and an *intermediate* suture passed between these, through the

* When the peritoneal surfaces of the intestine are held in apposition by this suture, adhesion occurs in remarkably short time. In January, 1887, I was called in consultation in a case of suspected volvulus. Upon opening the abdomen, it was found impossible to untwist the loop without puncture and evacuation of the contents of the greatly distended gut. The opening, one fourth of an inch long, was closed by four Lembert sutures at 11:30 a. m. At 3 p. m. the patient died. On autopsy, not only had well-marked adhesion taken place, but the silk threads were with difficulty recognized, being hidden beneath the inflammatory exudation.

substance of the mesentery and down into the strip of intestines which here is uncovered by peritonæum. Extra care must be taken to see that this part of each end of the cylinder is in perfect coaptation. The sutures are now inserted for the remainder of the apposing surfaces. The Lembert and intermediate sutures alternate through the entire circumference, and should be one eighth of an inch apart. The mucous or Czerny sutures should be from one fourth to three eighths of an inch apart. The relative proportion of these sutures is shown in Fig. 6. It is evident that while the Czerny suture is tied leaving the knot within the cavity of the intestine for the first part of the operation, the last few threads must be tied leaving the knot imbedded betweeen the mucous and mus-

Fig. 6.—Schematic. Section of intestine. [Showing the proportion of each form of suture, and their distance apart. *l*, Lembert; *i*, intermediate sutures alternating; *c*, Czerny sutures. (Natural size.

cular layers of the wall. In applying the sutures the plan followed was first a Czerny, then a Lembert about over this, next an intermediate, another Lembert, and after this a second Czerny suture, and so on. In other words it was necessary to insert the mucous suture before the superficial sutures had quite reached that point.

All of the threads should be cut off close to the knot.

In this operation I had to leave the space between the sutures on the upper end of the gut a little wider than on the lower, for the diameter of the efferent tube was considerably smaller than that of the afferent portion. The intervening space was a flush one-eighth of an inch on one side and a scant one-eighth of an inch on the other. When the sutures were all in, the constricting tapes were removed. The gut immediately filled with gas. To the surprise of all present, the intestine below the line of suture instantly expanded to a size equal to that of the portion above the line of union. That the wound was tightly closed was demonstrated by forcing the contents of the intestine from opposite directions toward the sutures. No gas escaped.

The appearance after the tapes were removed is shown in Fig. 7. At intervals of about five minutes during the operation, a small quantity of warm Thiersch solution was poured over the exposed intestine. The warm Thiersch towels upon which it rested, were changed every ten or fifteen minutes. No fluid was allowed to get into the abdominal cavity. Finally the intestine was carefully washed with this solution, and returned into the cavity of the peritonæum.

Fig. 7.—Showing the line of sutures in the mesentery and around the intestine.

It was now necessary to deal with the ring of intestine which occupied the femoral opening, and which led from the abscess into the abdominal cavity. Two strong silk threads were passed entirely through the opposing walls of this rim of intestine and tied so as to bring the edges well together. I then passed a silver probe from the hernial abscess cavity up through the femoral canal, and through the ring of adhering intestine between the two silk threads, until the end of the probe projected a half inch into the cavity of the abdomen. The ends of both threads were tied to the probe, and this withdrawn, bringing the sutures out through the saphenous opening. By making strong and continuous traction on these, the mucous membrane was everted, the peritoneal surfaces brought in contact, and the femoral opening closed. This procedure effected a radical cure of the hernia.

The wound in the parietal layer of peritonæum was closed by catgut sutures, introduced as in the Lembert suture. The abdominal incision was closed with silver sutures, which included all the tissues down to (but not touching) the peritonæum. For the prevention of ventral hernia after laparotomy, it is very important to include the fascia and aponeuroses of the muscles in the silver sutures. A Neuber's bone-drain was inserted. The abscess and sinus were packed with iodoformized gauze.

The operation lasted four hours. The patient rallied well, and was kept quiet with suppositories of opium. She was kept on the back, and was not permitted to move body, legs, or arms for ten days. The diet was milk, beef tea, and whisky in small quantities.

October 23d, 6 a. m., fourteen hours after operation, temperature 99° F. Patient vomited at 4:30 a. m.

24th.—Pulse 120, temperature 99° to 100°.

25th.—Pulse 100, temperature 99.6°. Patient comfortable. Slept well.

26th.—The pulse and temperature were the same.

27th.—Pulse 80 to 100, temperature 98.4° to 99.6°.

28th.—Pulse 100, temperature 99° to 100°.

29th.—Pulse 100 to 106, temperature 99.2°.

On this the sixth day the silk threads come away under the continuous traction of the elastic ligatures attached to them. The wire sutures were also removed. Wound of incision united throughout. Bowels moved; stool of normal consistence.

30th—Pulse 94 to 100, temperature 99.2° to 100.2° F. Bowels moved again; stool normal. Opium discontinued.

The subsequent history contains nothing of interest. The patient steadily gained her strength On November 20th she sat up in bed, and on December 3d was walking about the ward. She is now fully restored and attending to her duties. There is no sign of obstruction or interference with the functions of the alimentary canal, and the hernia is at this date radically cured. The great emaciation of the patient at the time of operation, and the fact that within hatf an inch of the opening into the abdomen there was a large abscess cavity, may be mentioned as the two conditions which rendered the prognosis grave.

The treatment of strangulated hernia with gangrene of the intestine may be considered under three methods:

1. Establishing a permanent fæcal fistula at the seat of gangrene.

2. Immediate exsection of the gangrenous portion of the gut, reunion of the ends by suture, and return of the loop.

3. Temporary fistula, followed, after an interval of some days, by laparotomy, excision, and suture.

To the first method may be consigned subjects so feeble that no operative procedure is justifiable.

As to whether exsection should be made at once or postponed after a free discharge through the fistula has been established must be determined by the condition of the individual at the time of operation. If the patient is well nourished, and if the anæsthetic is well borne, it will be advisable to relieve the strangulation, and through the hernial opening draw out the gut until five or six inches of sound intestine above and below the gangrenous spot are in sight, remove the dead portion, and unite the ends at once. This is a much simpler operation than when an additional opening through the abdominal wall is required.

In most cases, however, it will be found that the condition of the patient is not favorable for immediate exsection. Shock is almost always severe, and not infrequently fatal, when the constriction has been so severe or lasted long enough to produce gangrene. In such cases the plan carried out in the case just detailed should be followed.

Finally, the subject of intestinal suture is one of such vast importance that too much stress can not be laid upon the necessity for a thorough preparation for the operation. In the careful application of this procedure to penetrating wounds of the intestines, to exsection of gangrenous portions of the canal as the result of hernia, volvulus, intus-susception, and in the removal of malignant neoplasms and strictures, many lives may be saved which, under the teaching of former years, were left to die without surgical interference. The difficulties of the operation are great, and the time required in exsection dangerously long, unless the surgeon has had sufficient practice to enable him to work rapidly and safely. I would advise those who are willing to undertake this procedure to perfect themselves in the various sutures upon the cadaver, or preferably upon living animals. I was deeply impressed with the importance of this in my own case, for, notwithstanding that I had done this operation upon the cadaver about ten times, four hours were occupied in the case which forms the subject of this paper.

In penetrating wounds of the abdominal wall, the argument in favor of operative interference may be briefly stated as follows: 1. The enlargement of a wound sufficiently to demonstrate that it does or does not open into the cavity of the peritonæum is a simple procedure, and practically without danger. 2. A wound of the peritoneal cavity left without surgical interference is always attended with great danger, either from hæmorrhage immediately or from peritonitis at a later period. 3. If the alimentary canal is opened, death is almost inevitable; the few recorded cases of recovery form such an infinitesimal proportion of the whole that they should carry no weight against interference.—*New York Medical Journal.*

## Figures and Tables

**Fig. 1. f1:**
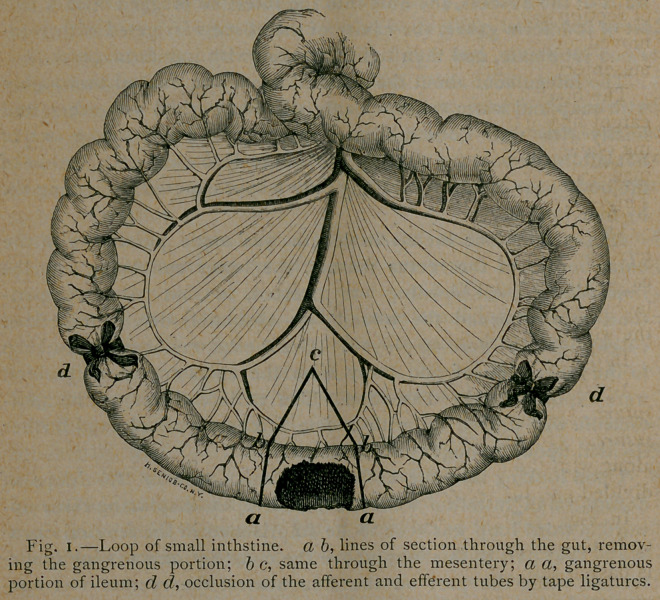


**Fig. 2. f2:**
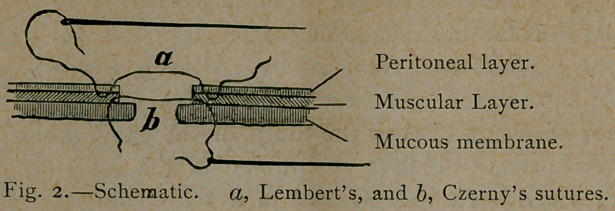


**Fig. 3. f3:**
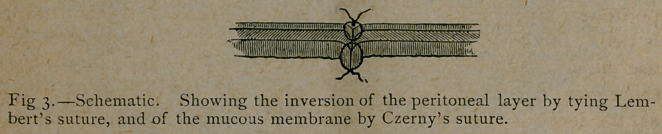


**Fig. 4. f4:**
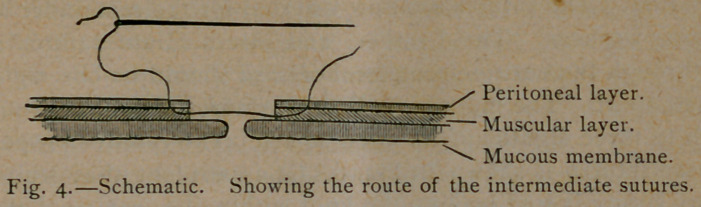


**Fig. 6. f5:**
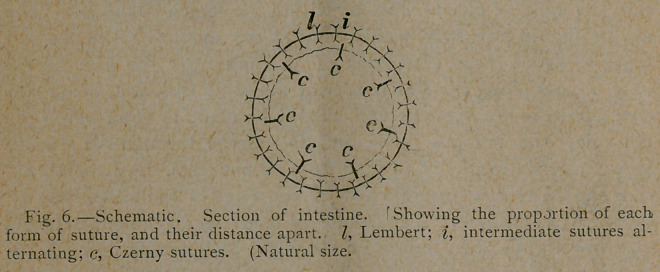


**Fig. 7. f6:**